# Metabolomic Analysis of the Effects of Cysteamine Zinc on the Composition and Amino Acid Profile of Mare’s Milk

**DOI:** 10.3390/life16060983

**Published:** 2026-06-11

**Authors:** Fan Yang, Yumei Ma, Xiaobin Li, Xinkui Yao, Kailun Yang, Caidie Wang

**Affiliations:** 1 Xinjiang Key Laboratory of Horse Breeding and Exercise Physiology, College of Animal Science, Xinjiang Agricultural University, Urumqi 830052, China; yangfan312au@163.com (F.Y.); 15109383544@163.com (Y.M.); lxb262819@163.com (X.L.); 19193552979@163.com (X.Y.); 2 Xinjiang Herbivore Nutrition Laboratory for Meat & Milk, College of Animal Science, Xinjiang Agricultural University, Urumqi 830052, China; 18723181520@163.com

**Keywords:** cysteamine zinc (CS-Zn), lactation performance, metabolites, amino acid metabolism

## Abstract

This study aims to investigate the effects of cysteamine zinc supplementation on milk production, composition, amino acid profile, and metabolites in mares. Building on prior experimental findings, a dose of 7 mg/kg body weight of CS-Zn was selected for the experimental group, which was compared with a control group. Milk samples were collected at various time points, and milk yield was recorded each time. Routine analysis of milk components, as well as the determination of milk metabolites and amino acids, were performed. The results indicated that, compared to the control group, the experimental group exhibited increases in milk yield and the content of milk fat, lactose, and non-fat solids (*p* < 0.05), with an extremely significant increase in milk protein (*p* < 0.01). Conversely, the levels of L-glutamine and L-proline in milk were significantly reduced (*p* < 0.05). Metabolomic analysis revealed that differentially expressed metabolites were enriched in pathways such as ABC transporters, D-aminoadipate metabolism, aminoacyl-tRNA biosynthesis, and protein digestion and absorption. Notably, milk metabolites including cAMp, biotin, and taurine showed a tendency to be upregulated, while oxoglutaric acid, methionine, and diacetyloxyxanthone were downregulated. Based on evidence from the literature other species, it is speculated that CS-Zn supplementation may be associated with alterations in endocrine and amino acid metabolism pathways, potentially influencing lactation performance in mares. However, because no hormones were directly measured in this study, such a mechanism remains speculative and requires direct experimental validation.

## 1. Introduction

Milk protein is a critical determinant of the nutritional quality of dairy products. It provides essential amino acids for growth in young animals and serves various physio-logical roles, including the production of bioactive peptides and immune system enhancement [1,2]. Protein composition varies among different livestock species. In cow’s milk, casein constitutes approximately 80% of the total protein making it ideal for cheese production [3,4]. Milk protein is a critical determinant of the nutritional quality of dairy products. It provides essential amino acids for growth in young animals and serves various physiological roles, including the production of bioactive peptides and immune system enhancement [1,2]. Protein composition varies among different livestock species. In cow’s milk, casein constitutes approximately 80% of the total protein, making it ideal for cheese production [3,4]. The protein profile of mare’s milk is notably similar to that of human milk, with a whey protein to casein ratio of approximately 1:1, in contrast to cow’s milk, where casein accounts for about 80% of the total protein [5,6]. Due to its low allergenicity, mare’s milk is considered a premium raw material for hypoallergenic dairy products and is also a potential alternative for infants with milk protein allergies [7]. However, mare’s milk contains a lower overall protein content compared to cow’s milk, particularly essential amino acids such as arginine, methionine, and histidine. This limitation hinders the full utilization of its nutritional potential [8]. Therefore, nutritional interventions aimed at optimizing the amino acid profile of mare’s milk could enhance its protein nutritional value.

In livestock production, the use of functional feed additives to regulate lactation performance and milk quality is a widely adopted nutritional strategy. Cysteamine zinc (CS-Zn), a dual-function additive combining cysteamine (CS) and zinc (Zn), offers notable advantages in animal metabolism [9]. CS is a bioactive peptide involved in coenzyme A composition, with antioxidant and anti-inflammatory properties that may contribute to animal health, and may enhance the quality of animal products [10]. Previous studies have indicated that CS has been suggested to reduce somatostatin levels in certain animal models [9], potentially influencing growth hormone (GH) secretion indirectly. However, direct evidence in mares is lacking; therefore, any role of CS in GH secretion or protein deposition in mares remains speculative. Zn, as a cofactor for numerous essential enzymes is directly involved in protein and nucleic acid synthesis, as well as cell division and proliferation [11]. Drawing on findings from other species, it is speculated that CS-Zn supplementation may be associated with alterations in the GH/SS axis, which may be related to changes in lactation performance. However, because GH and somatostatin were not directly measured in the present study, this potential mechanism remains speculative and requires direct experimental validation. Yang et al. [12] conducted a study by supplementing lactating dairy cows with CSH. The results showed that at a supplemental dose of 30 g/d, the growth hormone concentration was significantly higher than that in the control group (*p* < 0.05), while the differences in growth hormone levels at other doses were not significant. Wang et al.’s [13] study involving mid-lactation Holstein dairy cows CS-Zn supplementation led to significantly reduced plasma somatostatin levels and elevated GH content. Furthermore, supplementation of 20 g/(d·cow) CS in lactating Holstein dairy cows resulted in significantly higher milk production (*p* < 0.05), as well as fat content and protein content (*p* < 0.01) compared to the control group. Fan et al. [14] have also conducted research showing that supplementing CS-Zn in lactating sows can has also been shown to improve feed intake milk production and promote piglet growth. However, no study has yet integrated metabolomics and amino acid profiling to assess the effects of CS-Zn on mare milk composition. Unlike previous research in dairy cows and sows, this study focuses on mares and provides, for the first time, a comprehensive analysis of milk metabolites. All mechanistic interpretations are hypothesis-generating and require direct experimental validation, including hormone assays and mammary epithelial cell studies.

Therefore, this experiment selected Yili mares in lactation as the research subjects to investigate the effects of CS-Zn supplementation on the milk production composition under consistent feeding conditions. Additionally, metabolomics was employed to analyze overall changes in milk metabolites and amino acid metabolism. This study aimed to investigate the associations between CS-Zn supplementation and changes in milk production, milk composition, amino acid profiles, and milk metabolites in lactating mares, thereby providing preliminary evidence for future studies on the nutritional regulation of mare milk quality.

## 2. Materials and Methods

### 2.1. Experimental Design and Treatment

This study was conducted from June to October 2023 at the Kuudel Grassland of Zhaosu Farm in Zhaosu County, Yili Kazakh Autonomous prefecture. The experiment spanned 100 days, including a 10-day pre-feeding period and a 90-day main feeding period. All experimental procedures were approved by the Committee for the Welfare and Ethics of Laboratory Animals at Xinjiang Agricultural University (Approval Number: 2021092).

The data for this study were derived from previous research [15]. Twenty-four healthy lactating mares, with similar calving dates and an average body weight of 395.50 ± 28.60 kg, were randomly divided assigned to four groups, each consisting of six mares. The groups were as follows: control group (0 mg/kg·BW CS-Zn), experimental group I (3 mg/kg·BW), experimental group II (5 mg/kg·BW), and experimental group III (7 mg/kg·BW). All mares were kept under identical grazing conditions including grazing time, drinking time, milking time, and grazing pasture. The supplementation dosages were based on the study by Wang et al. [16], and the best dosage group (7 mg/kg·BW) along with the control group were selected for further in-depth analysis. It is important to note that only the control and the highest dose (7 mg/kg) groups were selected for the present analysis. The two intermediate dose groups (3 and 5 mg/kg) were excluded because preliminary data analysis revealed that they did not induce significant changes in milk yield, milk composition, or metabolite profiles compared to the control group, and no clear dose-response trend was observed. Thus, their biological relevance was limited. Focusing on the control and the optimal dose (7 mg/kg) allowed us to concentrate on the most biologically contrasting groups, a common strategy in exploratory metabolomic studies to avoid dilution effects. Nevertheless, the exclusion of intermediate doses precludes a complete dose-response assessment, which is a major limitation of this study. Future studies should include all dose groups in a systematic dose-response design.

Each day at 08:30, the mares and their foals were herded from the grazing pasture to the milking area. The mares in each group were administered the corresponding dose of CS-Zn in edible glutinous rice capsules (2.3 cm long, 0.8 cm in diameter), which were placed into their mouths to ensure ingestion At 09:00, the mares were separated from their foals, marking the start of the milking period, which lasted until 17:00. During this 8-h period, the mares were tethered and milked every 2 h, while the foals were kept apart and did not drink the mares’ milk. At 17:00, after the last milking session, the mares and foals were reunited and returned to the grazing pasture. For the remaining 16 h, the mares and foals grazed freely, with the mares eating and the foals nursing as desired. Despite the standardized milking and grazing protocols, factors such as ambient temperature, pasture quality fluctuations, and individual mare temperament were not fully controlled, which may have contributed to variability within the groups.

### 2.2. Pasture Grass, Plasma, Feces and Mare’s Milk Sample Collection

During the experimental period, fecal and forage samples from the mares were collected on the 28th and 30th days of each 30-day interval and immediately stored at −20 °C. After thawing at room temperature, the samples were mixed to calculate the dry matter intake (DMI) of forage by the mares. The acid-insoluble ash (AIA) method was employed for this purpose, which has been validated for use in grazing ruminants and horses in previous research [17]. However, it is acknowledged that this method assumes complete recovery of AIA and constant digestibility, which may not be entirely accurate. Although the AIA method is widely used, its expected error magnitude in horses is approximately 5–10% compared to total fecal collection; future studies should consider using indigestible markers such as n-alkanes for more accurate estimates.

Therefore, intake estimates should be interpreted as approximate.

The calculation formula is as follows: TDI $=\frac{F\;\times \;a}{b}$.

In this formula: TDI represents the dry matter intake of the experimental mare (kg·day^−1^·mare^−1^), F represents the dry matter excretion of the experimental mare per day (kg·day^−1^·mare^−1^),a represents the dry matter AIA content in the feces (%), and b represents the dry matter AIA content in the forage consumed by the mare (%). After calculation, the average DMI of the experimental mares was determined to be 12.98 ± 2.25 kg. The forage types in the grazing pasture included cocksfoot, smooth bromegrass, prairie needlegrass, alfalfa, prairie rough ryegrass, and various weeds. The types and nutritional levels of the mixed forage in the pasture are presented in Table 1.

During the trial period, the milk yield of each mare was recorded every other day during 8 h of tethering. Using the 8-h milk yield, the 24-h yield was estimated, and the average milk production over a 30-day period was calculated. On days 0, 30, 60, and 90, 100 mL of milk, 10 mL of plasma, and 50 mL of feces were collected from each mare and stored at −80 °C for later analysis.

### 2.3. Milk Composition Analysis

The MASTER ECO milk component analyzer (Instrument Series: 38,489) was used to measure the milk protein, fat, lactose, and non-fat solids in the milk samples.

### 2.4. Analysis of Amino Acid Metabolism in Mare’s Milk

For the preparation of samples, 50 μL of the milk sample was pipetted into an Ep tube. Then, 91 μL of distilled water, 100 μL of 0.15% sodium deoxycholate (DOC), and 4 μL of 100 μg/mL internal standard solution (Lys-d4/Try-d5/Gln-d4) were added. The mixture was vortexed and sonicated for 10 min (at 5 °C, 40 kHz). Afterward, 5 μL of 10 M trichloroacetic acid (TCA) was added, mixed by vertexing, and precipitated at freezing temperature for 10 min. The sample was centrifuged at 4 °C and 14,000 rcf for 10 min, and 50 μL of the supernatant was transferred to a new tube and mixed with 350 μL of water. The final solution was vortexed and filtered through a 0.2 μm pTFE filter membrane (Biotage) before LC-MS analysis.

Chromatographic conditions: AdvanceBioMS SpentMedia (2.1 × 50 mm, 2.7 µm), column temperature set at 40 °C, and injection volume of 1 μL. Mobile phase A 0.1% formic acid, 10 mM ammonium formate 95% water; mobile phase B 0.1% formic acid, 10 mM ammonium formate, 95% acetonitrile.

Data analysis was performed using AB Sciex quantitative software OS, applying default parameters for automatic identification and integration of each ion fragment, with manual verification as necessary. A linear regression standard curve was generated, plotting the mass spectrometry peak area of the analyte as the vertical axis against the analyte concentration as the horizontal axis. Sample concentrations were calculated by substituting the mass spectrometry peak area of the analyte into the linear equation.

### 2.5. Analysis of the Metabolome of Mare Milk

A volume of 100 μL sample of mare’s milk was placed in an Ep tube, followed by the addition of 400 μL of an 80% methanol-water solution. The mixture was vortexed and incubated on ice for 5 min and then centrifuged at 15,000× *g*, 4 °C for 20 min. A portion of the supernatant was then transferred and diluted with mass spectrometry-grade water to a methanol concentration of 53%. After a second centrifugation at 15,000× *g*, 4 °C for 20 min, the supernatant was collected and injected into the LC-MS for analysis. For the QC sample equal volumes from each experimental sample were combined. The blank sample was prepared by replacing the experimental sample with a 53% methanol-water solution. The pre-treatment of both the QC and blank samples followed the same procedure as the experimental sample.

Chromatographic conditions included a HypersilGold C18 column: at a temperature of 40 °C, with a flow rate of 0.2 mL/min. Mobile phase A consisted of 0.1% formic acid, while mobile phase B was methanol. The scan range was *m*/*z* 100–1500. The ESI source settings were as follows: spray voltage at 3.5 kV, sheath gas flow rate at 35 psi, auxiliary gas flow rate at 10 L/min, ion transmission tube temperature at 320 °C, ion import RF level at 60, auxiliary gas heater temperature at 350 °C, and polarity set to both positive and negative; MS/MS secondary scans were conducted in a data-dependent manner.

The raw data files were imported into the CD 3.3 search software for processing. Simple screening of parameters such as retention time and mass-to-charge ratio was conducted for each metabolite. Peak area correction was performed using the first QC sample to improve identification accuracy. The mass deviation was set to 5 ppm, with a signal intensity deviation of 30% and the inclusion of adduct ions for peak extraction. The peak area was quantified, and target ions were integrated. The molecular formula was predicted based on the molecular ion peak and fragment ions, which were compared with mzCloud (https://www.mzcloud.org/), mzVault and Masslist databases. Background ions were removed using the blank sample. The original quantitative results were standardized using the following formula: sample original quantitative value/(sum of sample metabolite quantitative values/sum of QC1 sample metabolite quantitative values). This calculation yielded relative peak areas. Compounds with a coefficient of variation (CV) in relative peak areas greater than 30% in the QC sample were excluded. Finally, the metabolite identification and relative quantification results were obtained. Data processing was carried out using the Linux operating system (CentOS version 6.6) and software R and python.

### 2.6. Metabolomics Data Processing and Quality Control

Raw LC-MS data were processed using Compound Discoverer 3.3 software, with automatic peak extraction, alignment, and retention time correction. Normalization was performed using total peak area normalization, followed by QC-based correction using the first QC sample as a reference. Metabolites with a CV greater than 30% in QC samples were excluded. This CV threshold is a standard in untargeted metabolomics to ensure data reproducibility. Metabolite identification was based on accurate mass (mass tolerance ≤ 5 ppm), retention time, and MS/MS fragmentation patterns. According to the Metabolomics Standards Initiative (MSI), putative annotations were assigned at confidence level 2 (putatively annotated compounds).

### 2.7. Statistical Analysis

All data are presented as mean ± standard deviation (SD). Each mare was considered an experimental unit for milk yield, milk composition, amino acid content, and metabolomics analysis. Routine statistical analyses were conducted using SPSS 27.0 software. Based on the results of previous experiments, this study focused primarily on comparing the control group (0 mg/kg) with the optimal dose group (7 mg/kg). For comparisons between the two groups, an independent samples *t*-test was applied to data with a normal distribution. A *p* value < 0.05 was considered statistically significant, and *p* < 0.01 was considered highly significant. For metabolomics data annotation, the KEGG database (https://www.genome.jp/kegg/pathway.html  accessed on 1 April 2026), HMDB database (https://hmdb.ca/metabolites accessed on 1 April 2026), and Lipid Maps database (http://www.lipidmaps.org/) were utilized. Multivariate statistical analysis, was performed using the metabolomics data processing software metaX. The data were transformed, followed by principal component analysis (PCA) and partial least squares discriminant analysis (PLS-DA), to calculate the variable importance in projection (VIP) value for each metabolite. PLS-DA models were validated using 200 permutation tests, and overfitting was assessed by the Q^2^ intercept (Q^2^ intercept < 0 indicates no overfitting). PCA was used exclusively for exploratory data visualization and not for statistical inference. Univariate analysis was performed using a *t*-test to calculate the statistical significance (*P* -value) of each metabolite between the two groups, and the fold change (FC) was calculated. Differential metabolites were selected based on the following criteria: VIP > 1, *p* < 0.05, and FC ≥ 2 or FC ≤ 0.5. All figures were generated using Origin 2024 software.

## 3. Results

### 3.1. Milk Yield and Milk Composition

The effects of CS-Zn supplementation on the milk yield and composition of lactating mares are summarized in Table 2. Compared to the control group, the CS-Zn group exhibited significant increases in milk yield, milk fat, lactose, and non-fat solids (*p* < 0.05), with a highly significant increase in milk protein (*p* < 0.01).

### 3.2. Untargeted Metabolomics Analysis

**Figure 1:**
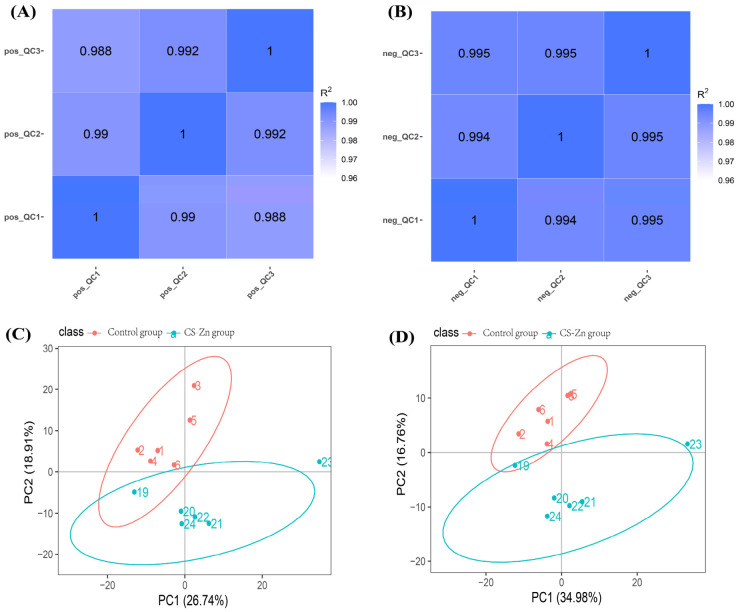

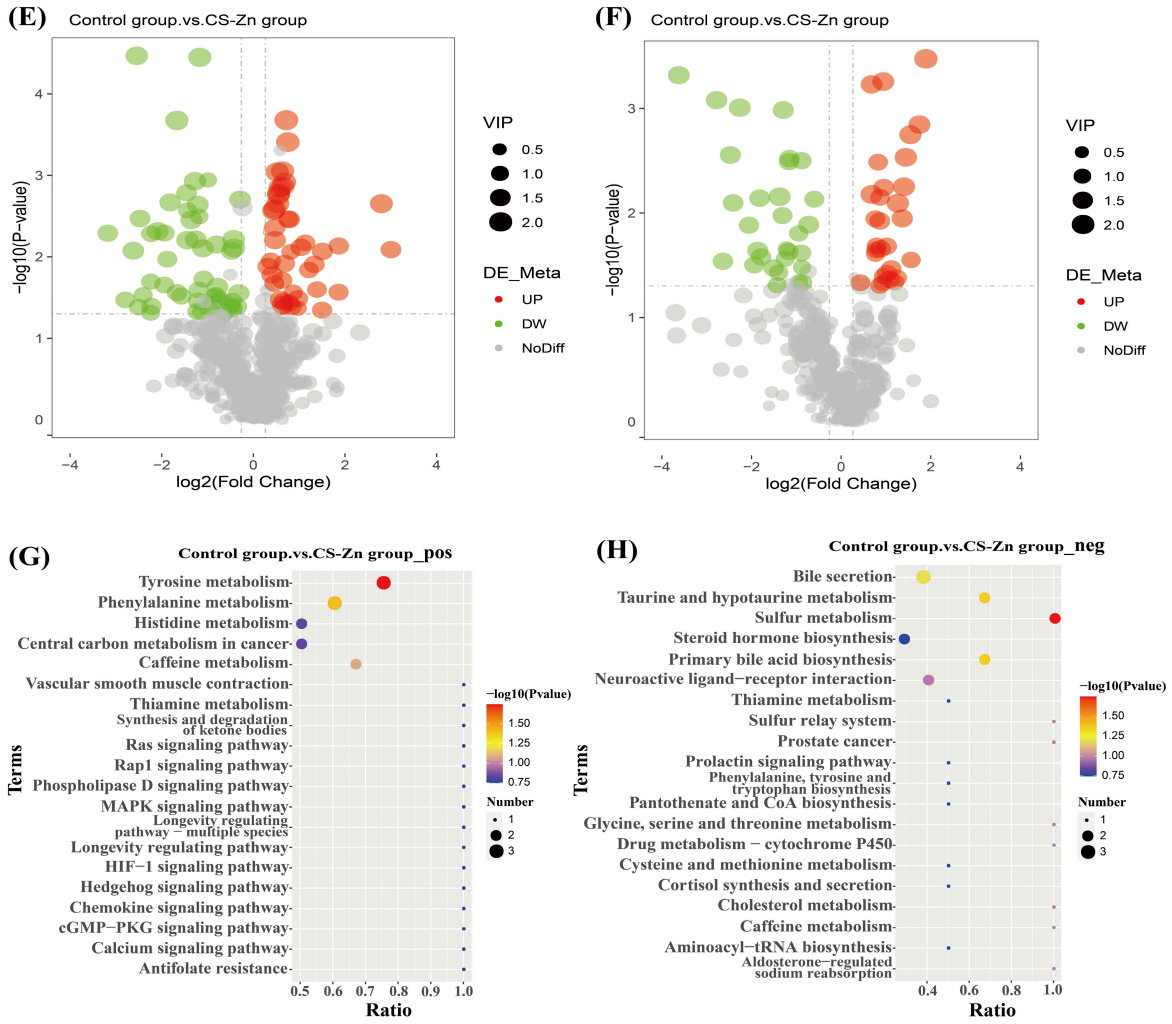
Effects of zinc cysteamineon metabolites in milk. (**A**) Correlation analysis of QC samples based on relative quantitative values of metabolites in positive ion mode. All correlation coefficients were greater than 0.99, indicating stable detection and high data quality. (**B**) Correlation analysis of QC samples based on relative quantitative values of metabolites in negative ion mode. All correlation coefficients were greater than 0.99, indicating stable detection and high data quality. (**C**) PCA analysis in positive ion mode: PC1 (26.74%) and PC2 (18.91%). Red: control group; blue: CS-Zn group (*n* = 6 per group). (**D**) PCA analysis in negative ion mode: PC1 (34.98%) and PC2 (16.76%). Red: control group; blue: CS-Zn group (*n* = 6 per group). (**E**) Positive ion mode, volcano plot of differential metabolites showing differentially expressed metabolites between the control group and the CS-Zn group. The x-axis represents log_2_(fold change), and the *y*-axis represents –log_10_(*p*-value). The size and color of the dots indicate VIP values (0.5–2.0). Screening criteria: VIP > 1, FC ≥ 2 or FC ≤ 0.5, and *p* < 0.05. Colors distinguish up-regulated (UP), down-regulated (DW), and non-significant metabolites. (**F**) Negative ion mode; volcano plot of differential metabolites, showing the same content as (**E**), reflecting the differential expression of metabolites between the two groups. (**G**) Positive ion mode; KEGG pathway enrichment analysis. The x-axis represents the enrichment ratio, and the y-axis represents –log_10_(*p*-value). (**H**) Negative ion mode; KEGG pathway enrichment analysis. The content is the same as that shown in (**F**). *p*-values are uncorrected for multiple comparisons; due to the exploratory nature of the study, they are provided only as descriptive statistics.

As presented in Figure 1A,B, the Pearsoncorrelation coefficients between the QC samples are all close to 1, demonstrating excellent stability throughout the detection process and high data quality, which meets the requirements of the subsequent metabolite analysis. The results from the PCA reveal clear separation between the control and experimental groups on the PCA plots for both positive (Figure 1C) and negative (Figure 1D) ions, showing separation between groups in the metabolomic profiles. The volcano plot (Figure 1E) in the cationic mode shows 97 different metabolites, with 43 upregulated and 54 downregulated. In the anionic mode (Figure 1F), 60 different metabolites were identified, with 30 upregulated and 30 downregulated. KEGG pathway enrichment analysis for the identified metabolites in both positive and negative ion modes highlighted several important pathways. In the cationic mode (Figure 1G), the top pathways included: tyrosine metabolism (acetoacetic acid, catechin, L-tyrosine; *p* = 0.0178), phenylalanine metabolism (2-phenethylamine, phenyl acrylic acid, L-tyrosine; *p* = 0.0394), histidine metabolism(L-histidine, 1-methylhistidine; *p* = 0.1474), cancer related carbon metabolism (L-histidine, methionine; *p* = 0.1474), and caffeine metabolism (7-methylxanthine, paraxanthine; *p* = 0.0826). In the anionic mode (Figure 1H), pathway enrichment highlighted: bile secretion (valproic acid, taurine deoxycholic acid, hydrocortisone; *p* = 0.0809), taurine and sub-taurine metabolism (Taurine, L-cysteamine; *p* = 0.0510), sulfur metabolism (Taurine, L-cystamine; *p* = 0.0185), steroid hormone biosynthesis (Hydrocortisone, Estrone; *p* = 0.2560), primary bile acid biosynthesis (Taurine, Taurine-Deoxycholic Acid; *p* = 0.0510), and neuroactive ligand-receptor interaction (Taurine, Hydrocortisone, *p* = 0.1439).Metabolite identification was performed at confidence level 2 (putatively annotated compounds) according to the Metabolomics Standards Initiative (MSI), based on accurate mass (≤5 ppm), retention time, and MS/MS fragmentation patterns. Therefore, all functional interpretations derived from these metabolites require targeted validation. (Full KEGG enrichment results for positive and negative ion modes are provided in Supplement Materials Tables S1 and S2, respectively).

### 3.3. Amino Acid Content

The effects of CS-−Zn supplementation on amino acid contents in mare milk are also shown in Table 3. Among the 20 detected amino acids, the contents of L−glutamine and L−proline in the CS−Zn group were significantly lower than those in the control group (*p* < 0.05). For the remaining 18 amino acids, no significant differences were observed between the two groups (*p* > 0.05). (Complete individual amino acid data for each mare are available in Supplement Materials Table S3).

### 3.4. Targeted Amino Acid Metabolomics Analysis

**Figure 2:**
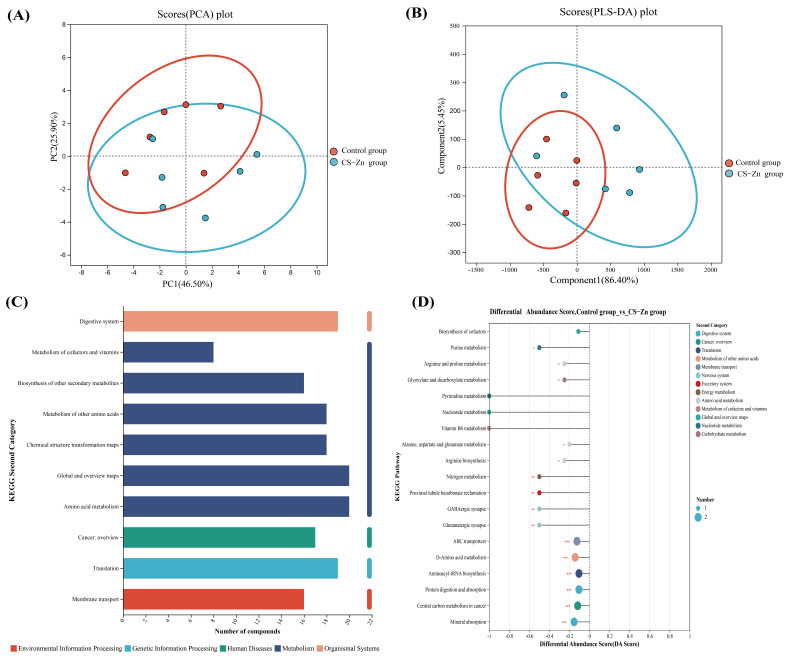

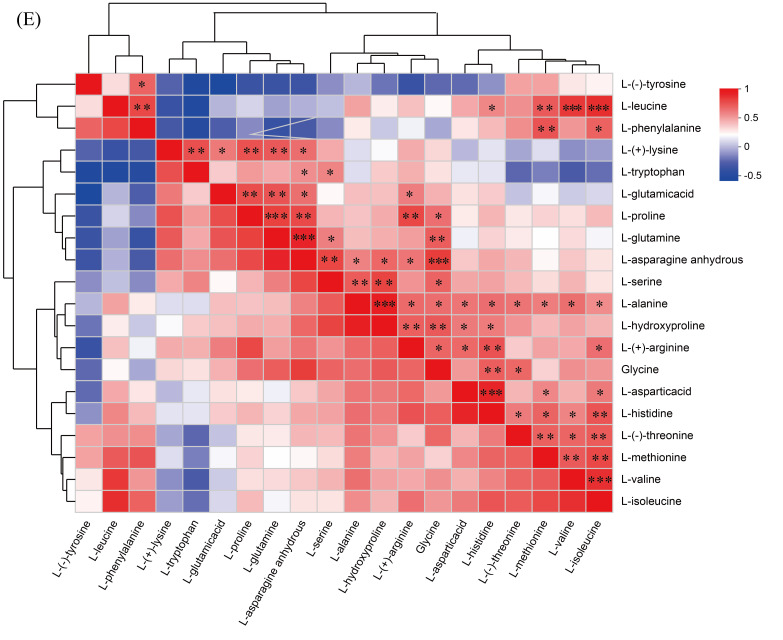
The effect of CS-Zn on amino acid metabolism in milk. (**A**) PCA (principal component) analysis; PC1 (46.50%) and PC2 (25.90%). Red: control group; blue: CS-Zn group (*n* = 6 per group); (**B**) Partial least squares discriminant analysis (PLS-DA); Component 1 (86.40%) and Component 2 (14.50%). Red: control group; blue: CS-Zn group. The first principal component was significant (R^2^ = 0.358, Q^2^ = 0.282), while the second principal component was not significant (Q^2^ negative, NS). Therefore, only the first principal component was retained in the model. After 200 permutation tests, the Q^2^ intercept was −0.12, indicating no overfitting. (**C**) KEGG pathway analysis. The x-axis shows the differential abundance score, the y-axis shows the related pathways, and different colors represent five functional categories; (**D**) KEGG pathway enrichment analysis, with bubble plot showing enriched KEGG pathways of differential metabolites. Bubble size represents the number of metabolites, and color indicates the significance of enrichment (* *p* < 0.05, ** *p* < 0.01, *** *p* < 0.001). (E)Metabolite correlation analysis, with Spearman correlation coefficients among differential metabolites shown. Red indicates positive correlation, blue indicates negative correlation, and color intensity reflects the strength of correlation. Correlation coefficients are displayed within each cell. Asterisks denote significance (* *p*< 0.05, ** *p* < 0.01, *** *p* < 0.001).

The effects of supplementary CS-Zn on amino acids in the milk of lactating mares are presented in Figure 2. The PCA results, shown in Figure 2A, reveal that the *x*-axis represents the first principal component, contributing 48.60% to the variance in the samples, while the *y*-axis represents the second principal component, contributing 8.90%. The PLS-DA results in Figure 2B indicate that PC1 (the first predictive principal component) explains 86.40% of the variance, while PC2 (the first orthogonal component) explains 5.45%. Figure 2C presents the KEGG pathway analysis, showing the distribution of annotated metabolites across various functional categories, including metabolism, genetic information processing, environmental information processing, organismal systems, and human diseases. Figure 2D displays the KEGG pathway enrichment analysis, which shows that the expression trends of all annotated differential metabolites in the pathways were decreased. The pathways with the highest number of differential metabolites include ABC transporters, D-Amino acid metabolism, aminoacyl−tRNA biosynthesis, protein digestion and absorption, central carbon metabolism in cancer, and mineral absorption. Figure 2E presents a correlation analysis of the metabolites. indicating positive correlations between isoleucine and leucine, isoleucine and valine and proline and arginine.

### 3.5. Correlation Analysis

**Figure 3:**
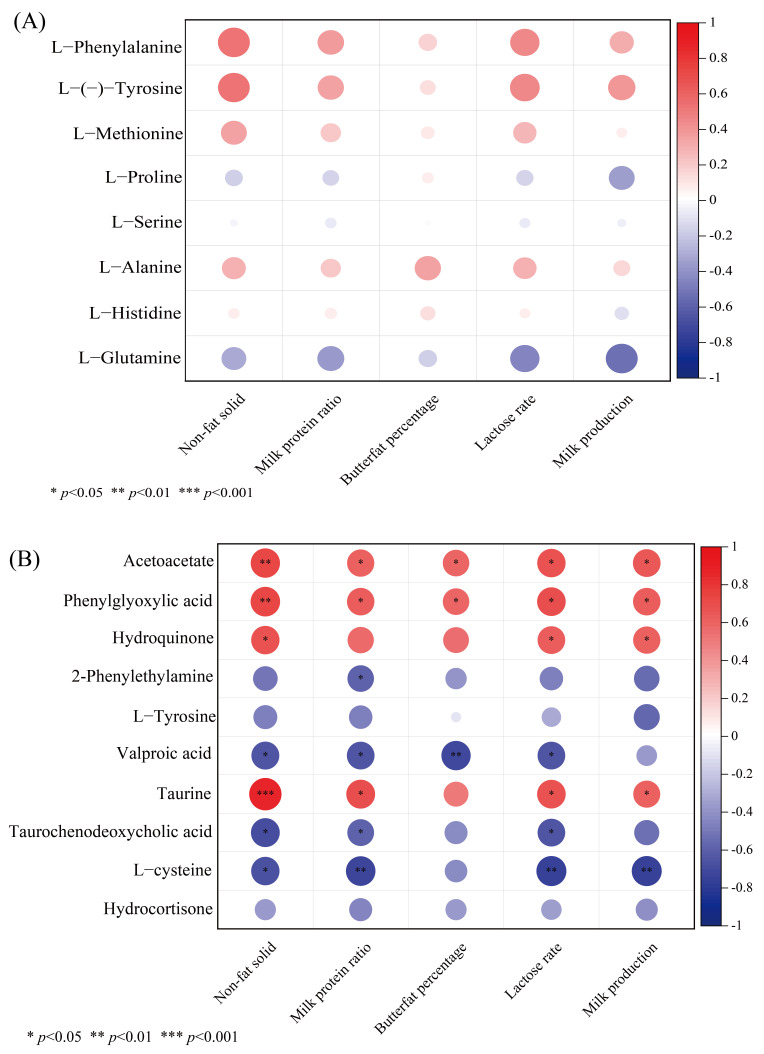
Correlation analysis correlation heatmap (Spearman). (**A**) Correlation analysis between amino acids and lactation performance; (**B**) Correlation analysis between differential metabolites and lactation performance. Red: positive correlation; blue: negative correlation; darker colors indicate stronger correlations. Asterisks denote significance levels (* *p*<0.05, ** *p* < 0.01, *** *p* < 0.001); no asterisk indicates *p* > 0.05.

All correlation analyses were exploratory in nature and do not imply causality. Correlation cannot establish causal relationships; the observed associations may reflect indirect relationships or confounding factors. These results should be considered hypothesis-generating. Following KEGG pathway enrichment analysis of lactation performance and differential metabolites in milk, the four metabolic pathways with the highest number of differential metabolites—tyrosine metabolism, phenylalanine metabolism, bile secretion, and taurine and hypo taurine metabolism—were selected for further correlation analysis (Figure 1G,H). By focusing on amino acid metabolism in milk, the KEGG pathway analysis(Figure 2D) reveals that the highest numbers of amino acids were detected in the pathways related to ABC transporters, D-amino acid metabolism, aminoacyl-tRNA biosynthesis, protein digestion and absorption, central carbon metabolism in cancer, and mineral absorption. Based on the results of differential metabolites in milk and amino acid content, L−phenylalanine, L−(+)−tyrosine, L−methionine, L-proline, L−serine, L−histidine, L−alanine, and L−glutamine were selected for correlation analysis. Figure 3A shows the correlation analysis between amino acids and lactation performance. None of the correlations reached statistical significance; the following observations are purely descriptive and should not be interpreted inferentially. Specifically, phenylalanine and tyrosine showed darker red colors with non-fat solids and lactose, suggesting a relatively strong positive correlation between these two amino acids and the aforementioned milk components. Figure 3B presents the correlation analysis between differential metabolites and lactation performance, with more definitive results, most of which reached significant levels. Acetoacetate and phenyl glyoxylic acid were significantly positively correlated with non-fat solids, milk protein, milk fat, lactose, and milk yield. Hydroquinone was significantly positively correlated with non-fat solids, lactose, and milk yield. Taurine was also significantly positively correlated with non-fat solids, milk protein, lactose, and milk yield. Thus, these differential metabolites were positively associated with improvements in lactation performance. However, these associations are exploratory and require validation in future studies.

## 4. Discussion

It is important to note that this study did not directly measure key hormones such as somatostatin and growth hormone (GH), nor did it assess mammary epithelial cell proliferation markers. Therefore, any mechanistic interpretations regarding endocrine regulation or mammary cellular processes should be considered speculative and hypothesis-generating. Direct experimental validation, including hormone assays and mammary epithelial cell studies, is required to establish causality.

In the present study, CS-Zn supplementation at 7 mg/kg BW was associated with increased milk yield, milk fat, lactose, non-fat solids, and milk protein in mares, accompanied by notable alterations in milk metabolite and amino acid profiles. These findings provide preliminary evidence that CS-Zn may be related to changes in mare milk composition and metabolic characteristics. However, the present results should not be interpreted as direct evidence that CS-Zn regulates lactation through the GH/SS axis.

Previous studies in other species have suggested that cysteamine may be associated with changes in somatostatin and GH levels [12,18], and GH has been reported to participate in nutrient partitioning, mammary gland development, and lactational regulation [19,20,21]. Cysteamine supplementation has also been reported to affect milk yield and mammary cell-related traits in dairy cows, dairy goats, and rats [12,22].Similarly, Lv et al. [23] observed that as the cysteamine dose increased, milk fat percentage, protein percentage, and protein yield followed a significant quadratic trend, first increasing and then decreasing. Based on these studies, it is speculated that CS-Zn may influence lactation-related metabolic regulation through pathways involving the GH/SS axis. However, because GH, somatostatin, and mammary epithelial cell proliferation markers were not measured in the present study, this potential mechanism remains speculative and requires direct validation. In addition, there are considerable differences in digestive physiology between horses and ruminants. Horses are hindgut fermenters, characterized by rapid gastric emptying, high enzymatic digestion in the small intestine, and extensive microbial fermentation in the large intestine [24,25], whereas ruminants rely on rumen fermentation. Furthermore, the whey-to-casein ratio in mare mill is approximately 1:1, in contrast to the casein-dominant composition of bovine milk [5,26].Therefore, the mechanisms reported in ruminants cannot be directly extrapolated to mares, and the findings of the present study should be validated in horse-specific studies.

Changes in milk composition are closely linked to the metabolic state of the mother, with small-molecule metabolites in milk serving as key indicators of both milk quality and maternal metabolism [27]. The metabolomics analysis in this study revealed that CS-Zn supplementation was associated with significant alterations in lipid and lipid-like metabolites, which corresponded to the observed increase in milk fat content. Further analysis highlighted the upregulation of cAMp, taurine, and biotin as key features. Elevated cAMp levels may be linked to enhanced milk secretion, aligning with the increase in milk yield [28]. The increased taurine level may be related to the role of cysteamine as a precursor for taurine biosynthesis [29,30]. However, because hormone concentrations and mammary lipid synthesis markers were not directly measured, the relationship among taurine metabolism, endocrine regulation, and milk fat synthesis remains hypothetical and requires further experimental validation. Biotin, a critical coenzyme involved in fatty acid, carbohydrate, and amino acid metabolism [31], may play a role in optimizing nutrient conversion into milk components. Because no direct measurements of somatostatin, GH, or mammary epithelial cell activity were performed in this study, the following interpretations are correlational and hypothesis-generating only. However, these interpretations remain speculative and are based on correlational data; causal mechanisms have yet to be directly validated and require further experimental investigation. Additionally, oxo adipic acid and methionine were detected as metabolites in mare milk. Oxo adipic acid, a product of essential amino acids like tryptophan and lysine, is involved in amino acid metabolism [32], while methionine, a sulfur-containing essential amino acid, plays a pivotal role in protein synthesis, and its deficiency impairs this process [33].

Amino acids serve as both the building blocks for milk protein synthesis and regulatory factors during lactation [34], with their metabolic changes closely linked to improvements in milk yield and composition. In mare milk, the whey-to-casein ratio is approximately 1:1 [35], and whey proteins are rich in limiting amino acids such as lysine and methionine [36], which are essential for human nutrition. In this study, the 20 detected amino acids exhibited differential changes: some essential amino acids (e.g., isoleucine, tryptophan, lysine, valine) increased, potentially providing more substrates for milk protein synthesis, while others (e.g., leucine, methionine, phenylalanine, threonine) decreased, possibly due to higher metabolic demands associated with increased mammary activity. Among the non-essential amino acids, only alanine and tyrosine increased, while all others decreased, with L-glutamine and L-proline showing significant reductions. The decreases in these two amino acids may reflect their increased utilization by the mammary gland for milk protein synthesis. In lactating mammals, amino acids are actively taken up by mammary epithelial cells and incorporated into casein and whey proteins. A decrease in free amino acid levels in milk could indicate enhanced protein synthesis or altered nitrogen balance. However, without direct measurements of nitrogen balance or amino acid flux analysis, this interpretation remains speculative and warrants further experimental validation. The KEGG pathway enrichment analysis revealed that the differential amino acids were primarily enriched in pathways such as ABC transporters, aminoacyl-tRNA biosynthesis, and protein digestion and absorption. ABC transporters facilitate the transmembrane transport of amino acids and other nutrients, while aminoacyl-tRNA biosynthesis is a critical step in protein synthesis. The enrichment of these pathways suggests that CS-Zn may enhance milk protein synthesis by promoting amino acid transport and improving protein synthesis efficiency. Notably, the marked increase in milk protein was accompanied by reductions in glutamine and proline, indicating that these amino acids may be preferentially utilized for milk protein synthesis or metabolized by mammary epithelial cells. Further correlation analysis revealed that certain metabolites (e.g., acetoacetate, phenyl glyoxylic acid) were positively correlated with milk fat, lactose, milk protein, and non-fat solids, while valproic acid was negatively correlated with milk components. Acetoacetate, a ketone body, is an important substrate for mammary energy metabolism and provides energy for the synthesis of milk fat, protein, and lactose [37]. phenyl glyoxylic acid may be involved in amino acid metabolism, with its level change reflecting protein metabolism and synthesis efficiency. Hydroquinone was positively correlated with milk protein. While hydroquinone is known for its antioxidant properties, its role in milk synthesis remains unclear, and this finding should be interpreted cautiously, requiring further experimental validation. Similarly, the negative correlation of valproic acid with several milk components is intriguing, but its biological significance in mare milk remains uncertain. However, direct experimental evidence is lacking, and these interpretations should be considered preliminary.

The small sample size (n = 6 per group) in this study may increase the risk of type II errors and potential overfitting in multivariate models (e.g., pLS-DA). Therefore, the results should be interpreted with caution. Additionally, the study did not measure somatostatin, GH, or mammary epithelial cell proliferation markers, making any mechanistic interpretations regarding endocrine regulation and cell proliferation speculative and aimed solely at hypothesis generation. Although two intermediate dose groups (3 and 5 mg/kg) were initially included in the experimental design, only the control group and the highest dose group (7 mg/kg) were compared. Additionally, the exclusion of the two intermediate dose groups (3 and 5 mg/kg) was based on their lack of significant effects on lactation performance and milk metabolites in our preliminary analysis. While this focus on extreme groups is methodologically reasonable for hypothesis generation, it limits our ability to draw dose-response conclusions. The exclusion of the intermediate doses precludes a complete dose-response assessment, preventing conclusions regarding the optimal dose and limiting the generalizability of the findings. Future studies should incorporate dose-response designs, larger sample sizes, direct measurements of relevant hormones and cell proliferation markers, and the validation of metabolite biomarkers across different mare populations.

## 5. Conclusions

In conclusion, supplementation with CS-Zn at 7 mg/kg BW was associated with increased milk yield, elevated milk fat, lactose, non-fat solids, and milk protein, along with changes in amino acid and metabolite profiles in mare milk. Based on evidence from the literature on other species, it is speculated that CS-Zn may influence the GH/SS axis; however, because no hormones were measured in this study, such a mechanism remains speculative and requires direct experimental validation. The exclusion of intermediate dose groups and the small sample size limit the generalizability of the results. Future studies should incorporate dose-response designs, direct hormone measurements, and larger sample sizes to validate these observations.

## Figures and Tables

**Table 1:** Nutritional composition of pasture on an air-dried basis (%).

Items	September
DM (%)	91.58
OM (%)	92.64
GE (MJ/kg)	17.64
Cp (%)	11.28
EE (%)	2.83
CF (%)	36.56
Ca (%)	0.87
*p* (%)	0.17

**Table 2:** The effect of CS-Zn on lactation performance.

Item	Control Group	CS-Zn Group	*p*
Milk yield (kg/d)	2.79 ± 0.25	3.52 ± 0.65	0.03
Lactose (%)	6.10 ± 0.08	6.38 ± 0.17	0.01
Milk fat (%)	0.88 ± 0.37	1.37 ± 0.23	0.02
Milk protein (%)	1.60 ± 0.05	1.84 ± 0.08	<0.001
Non-fat solids (%)	8.52 ± 0.16	9.06 ± 0.30	0.01

Note: Data are presented as mean ± SD (*n* = 6 per group). Comparisons between groups were performed using independent samples *t*-test. *p* < 0.05 indicates a significant difference, *p* < 0.01 indicates a highly significant difference. Control group: 0 mg/kg CS-Zn; CS-Zn group: 7 mg/kg CS-Zn.

**Table 3:** The effect of supplementary CS−Zn on the amino acid content in mare milk (µg/mL).

		Control Group	CS-Zn Group	*p*
Essential amino acids	L−Isoleucine	1.11 ± 0.27	1.10 ± 0.35	0.976
	L−Leucine	2.41 ± 0.67	2.97 ± 1.16	0.336
	L−Tryptophan	2.36 ± 0.47	1.85 ± 0.50	0.102
	L−(+)−Lysine	14.69 ± 4.88	10.33 ± 3.40	0.103
	L−Methionine	0.08 ± 0.03	0.09 ± 0.03	0.563
	L−phenylalanine	0.89 ± 0.24	1.27 ± 0.37	0.058
	L−(−)−Threonine	9.00 ± 2.48	9.31 ± 1.66	0.806
	L−Valine	3.55 ± 0.75	3.47 ± 1.17	0.892
	L−(+)−Arginine	16.43 ± 4.23	13.25 ± 4.38	0.230
	L−Histidine	10.14 ± 7.12	8.27 ± 6.28	0.640
Non-essential amino acids	L−Alanine	31.87 ± 5.23	32.31 ± 4.08	0.879
	L−proline	1.36 ± 0.32	1.01 ± 0.17	0.042
	L−Asparagine Anhydrous	24.31 ± 6.34	18.17 ± 4.42	0.080
	L−Aspartic Acid	9.96 ± 2.17	9.46 ± 1.68	0.861
	L−Glutamine	668.77 ± 288.97	343.05 ± 156.18	0.036
	L−Glutamic Acid	180.85 ± 65.11	150.43 ± 24.67	0.310
	Glycine	3.33 ± 1.01	2.34 ± 0.66	0.077
	L−Serine	20.01 ± 9.54	14.37 ± 8.71	0.310
	L−Hydroxyproline	2.36 ± 0.39	2.28 ± 0.27	0.649
	L−(−)−Tyrosine	0.38 ± 0.11	0.66 ± 0.34	0.104

Note: Data are presented as mean ± SD (*n* = 6 per group). Comparisons between groups were performed using independent samples *t*-test. *p* < 0.05 indicates a significant difference, *p* < 0.01 indicates a highly significant difference. Control group: 0 mg/kg CS−Zn; CS−Zn group: 7 mg/kg CS−Zn.

## Data Availability

All data generated or analyzed during this study are included in this published article.

## Supplementary Materials

The following supporting information can be downloaded at: https://www.mdpi.com/article/10.3390/life16060983/s1, Table S1: Nutritional composition of pasture on an air-dry basis (%); Table S2: The effect of CS-Zn on lactation performance; Table S3: The effect of supplementary CS-Zn on the amino acid content in mare milk (µg/mL).

## Abbreviations

The following abbreviations are used in this manuscript:

DM	Dry matter
OM	Organic matter
GE	Gross energy
Cp	Crude protein
EE	Ether extract
CF	Coarse fiber
